# First-principles investigation on electronic properties and band alignment of group III monochalcogenides

**DOI:** 10.1038/s41598-019-49890-8

**Published:** 2019-09-16

**Authors:** Chongdan Ren, Sake Wang, Hongyu Tian, Yi Luo, Jin Yu, Yujing Xu, Minglei Sun

**Affiliations:** 1 Department of Physics, Zunyi Normal College, Zunyi, Guizhou 563002 China; 2 College of Science, Jinling Institute of Technology, Nanjing, Jiangsu 211169 China; 3 School of Physics and Electronic Engineering, Linyi University, Linyi, Shandong 276005 China; 4 School of Materials Science and Engineering, Southeast University, Nanjing, Jiangsu 211189 China; 5 School of Mechanical Engineering, Southeast University, Nanjing, Jiangsu 211189 China

**Keywords:** Surfaces, interfaces and thin films, Electronic structure

## Abstract

Using first-principles calculations, we investigated the electronic properties and band alignment of monolayered group III monochalcogenides. First, we calculated the structural and electronic properties of six group III monochalcogenides (GaS, GaSe, GaTe, InS, InSe, and InTe). We then investigated their band alignment and analysed the possibilities of forming type-I and type-II heterostructures by combining these compounds with recently developed two-dimensional (2D) semiconducting materials, as well as forming Schottky contacts by combining the compounds with 2D Dirac materials. We aim to provide solid theoretical support for the future application of group III monochalcogenides in nanoelectronics, photocatalysis, and photovoltaics.

## Introduction

Group III monochalcogenides (M_III_Xs, where M_III_ represents a group III element and X represents a chalcogen), a family of monolayered semiconducting materials, have attracted much research interest in recent years^1–8^. M_III_Xs are semiconductors with moderate bandgaps which are sensitive to the number of layers in the material^9^. Their suitability for use as transistors^10^, sensors^11^, and photodetectors^12–16^ has been addressed in a number of studies. For example, Lei *et al*.^13^ fabricated an ultrathin InSe-based photodetector whose overall performance surpassed those of similar devices. Many researchers also investigated the effects of doping^17^, defects^18^, applied elastic strain^19^, and an external electric field^20^ on the electronic and optical properties of M_III_Xs. More importantly, many types of M_III_X have been synthesized^21^. All these investigations demonstrate that M_III_Xs can be an important category of 2D semiconductor materials for application in many fields.

In this paper, we report the results of our comprehensive investigation on the electronic properties and band alignment of M_III_Xs (M_III_ = Ga or In, X = S, Se, or Te; including GaS, GaSe, GaTe, InS, InSe, and InTe). More specifically, the structural parameters, band structures, and band edges were calculated for each material. We then explored the possibility of these materials forming type I, II, and III heterostructures with popular 2D semiconducting materials, including MoS_2_, MoSe_2_, WS_2_, WSe_2_, black phosphorene, blue phosphorene, arsenene, h-BN, g-GaN, and germanane; the results are reported here. In addition, we also report our analysis of the possibility to form Schottky contacts between M_III_Xs and 2D Dirac materials such as graphene and silicene. Our results will not only provide basic information on the properties of M_III_Xs, but also fundamental guidelines for future application of M_III_Xs.

## Calculation Details

We used density-functional theory with the Perdew−Burke−Ernzerhof functional^22^ and projector-augmented waves^23,24^ to treat the valence electrons as implemented in the Vienna Ab Initio Simulation Package (VASP)^25,26^. The hybrid Heyd−Scuseria−Ernzerhof (HSE06) functional^25^ was also selected to compute the electronic properties. The mixing parameter was set to 0.25, while the screening parameter was set to 0.2 Å^-1^. The zero-damping vdW-D3 correction proposed by Grimme^26^ was used to describe the long-range interaction. The energy cutoff for plane-wave expansion was set to 550 eV. A 21 × 21 × 1 *k*-point mesh with Monkhorst–Pack^27^ scheme was used to sample the first Brillouin zone. The tetrahedron methodology with Blöchl corrections^28^ was used for all the calculations, only except the Gaussian smearing methodology^29^ with a smearing of 0.01 eV was employed for band structure calculations. To avoid interaction between adjacent images, a relatively large vacuum space of 20 Å was inserted in the normal direction. After the fully relaxation, the force on each atom was less than 0.01 V/Å. All the calculations were performed in a spin-restricted manner.

## Results and Discussions

**Figure 1:**
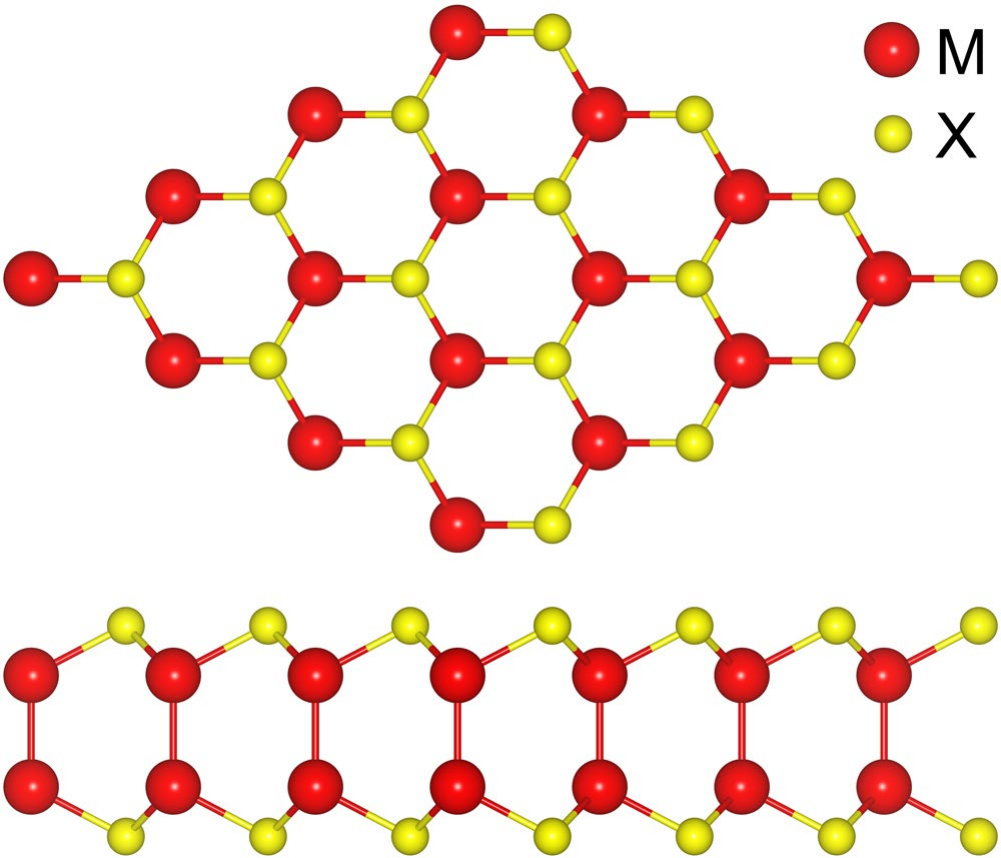
Crystal structure of M_III_Xs examined in this study: GaS, GaSe, GaTe, InS, InSe, and InTe.

The crystal structure of M_III_X is shown in Fig. 1. A M_III_X monolayer is formed by four covalently bonded atomic planes in an X–M_III_–M_III_–X sequence. The relaxed lattice constants of GaS, GaSe, GaTe, InS, InSe, and InTe are 3.62, 3.80, 4.12, 3.91, 4.06, and 4.36 Å, respectively. Meanwhile, the thicknesses of GaS, GaSe, GaTe, InS, InSe, and InTe are 4.65, 4.80, 4.99, 5.19, 5.37, and 5.56 Å, respectively.

**Figure 2:**
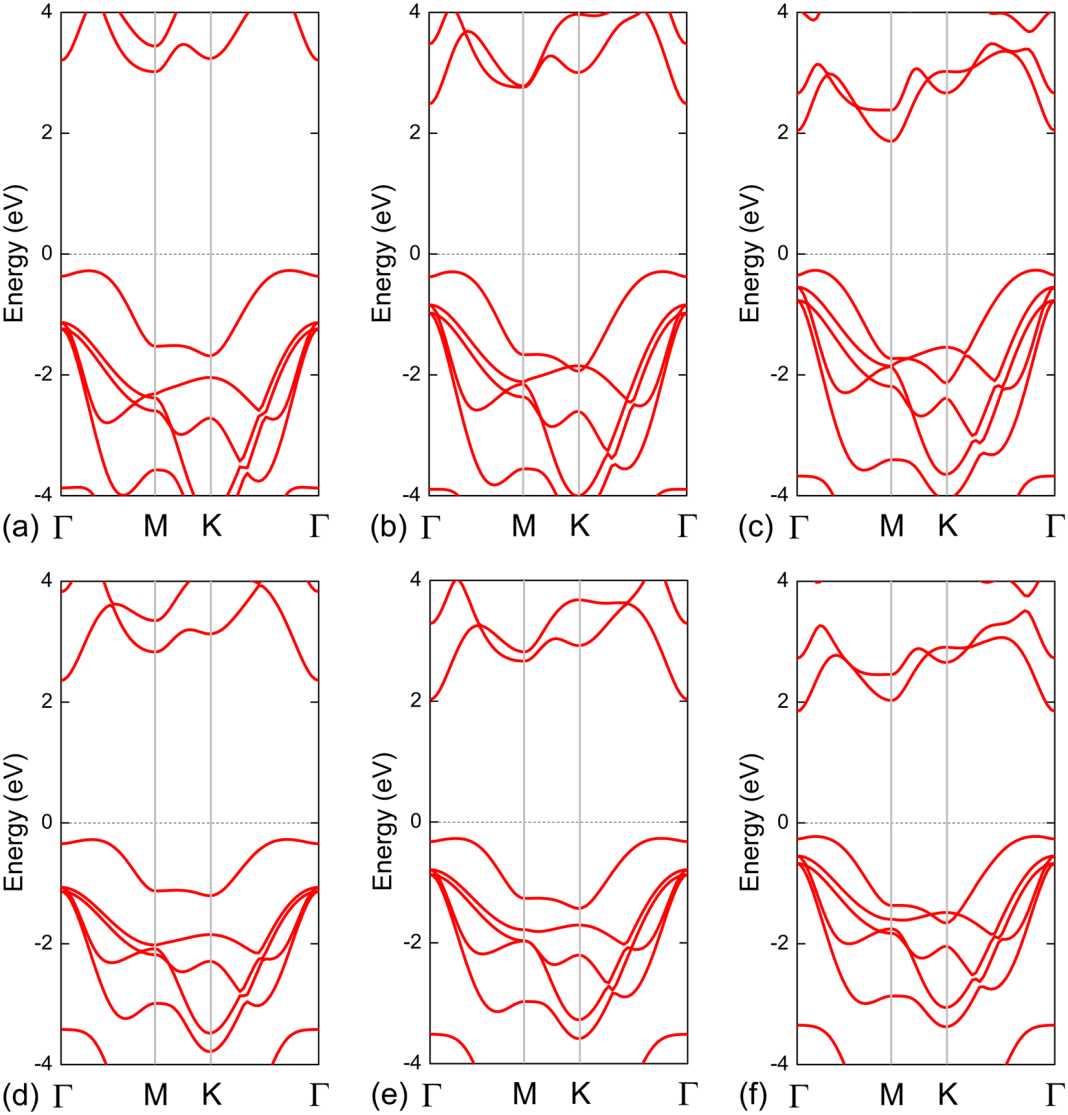
Band structures of (**a**) GaS, (**b**) GaSe, (**c**) GaTe, (**d**) InS, (**e**) InSe, and (**f**) InTe monolayers, obtained using the HSE06 functional. The black dashed line denotes the Fermi level.

The band structures of the M_III_Xs are shown in Fig. 2. All of the M_III_X monolayers are indirect-bandgap semiconductors. For each M_III_X monolayer, the valence-band maximum (VBM) is located between the Γ and M points; however, the positions of their conduction-band minimum (CBM) are slightly different. For GaS and GaTe, their CBM are located at their respective M points. Meanwhile, for GaSe, InS, InSe, and InTe, their CBM are located at their respective Γ points. Using the HSE06 functional, the calculated gap values for GaS, GaSe, GaTe, InS, InSe, and InTe monolayer are 3.29, 2.77, 2.13, 2.63, 2.30, and 2.07 eV, respectively.

We observed an interesting feature in the band structures of these M_III_Xs – band convergence. For all of the M_III_X monolayers, in addition to the VBM located at the Γ–M high-symmetry line, there is another valley in the valence band along the K–Γ high-symmetry line. The difference between the energies of these two valleys is only 4, 25, 34, 18, 18, and 3 meV for the GaS, GaSe, GaTe, InS, InSe, and InTe monolayers, respectively, which are all much lower than 52 meV (2*k*_B_*T*_300K_, i.e. twice the thermal energy at room temperature). This means that band convergence may occur in the valence band of GaS, GaSe, GaTe, InS, InSe, and InTe. When both valleys contribute to the total electrical conductivity (*σ*), the power factor (*P = S*^2^*σ*, where *S* presents the Seebeck coefficient) is considerably increased. Since the Seebeck coefficient is one of the main factors of a material’s ability to efficiently produce thermoelectric power, the band convergence in the GaS, GaSe, GaTe, InS, InSe, and InTe monolayers may allow them to be used as thermoelectric materials, as previously shown for MoS_2_^30–32^ and phosphorene^33^. Indeed, Tung *et al*.^34^ found that the maximum *P* of a p-type (and n-type) InSe monolayer can reach 0.049 (and 0.043) W/K^2^m at 300 K in the armchair direction.

**Figure 3:**
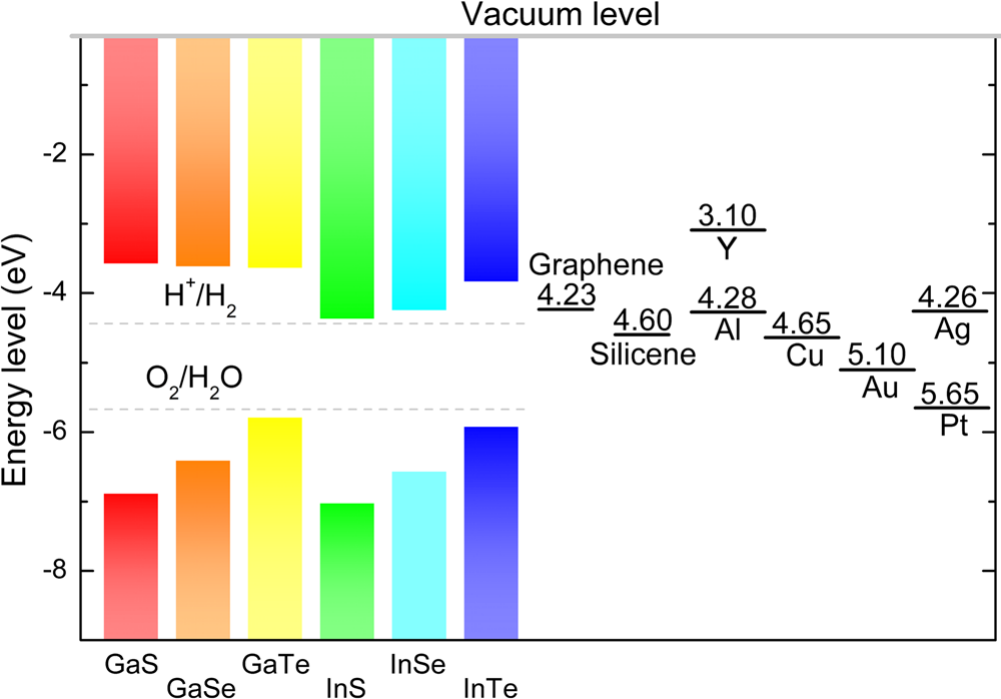
Band alignment of GaS, GaSe, GaTe, InS, InSe, and InTe monolayers. The energy of the vacuum level was set to zero. The work functions of graphene and silicene, as well as those of elemental Y, Al, Cu, Ag, Au, and Pt are also shown; the work functions of Y, Al, Cu, Ag, Au, and Pt were obtained from experimental data in^43^.

The band alignment of M_III_Xs is shown in Fig. 3. The energy levels of CBM and VBM were calculated with reference to the

vacuum level, while the vacuum level was determined through the calculation of the planar averaged electrostatic potential. The VBM values of GaS, GaSe, GaTe, InS, InSe, and InTe monolayers are −6.88, −6.40, −5.78, −7.02, −6.56, and −5.91 eV, respectively. Meanwhile, the CBM values of GaS, GaSe, GaTe, InS, InSe, and InTe monolayers are −3.59, −3.63, −3.65, −4.38, −4.26, and −3.84 eV, respectively. As reported in a previous investigation^35^, the reduction potential (${{\rm{E}}}_{{{\rm{H}}}^{+}/{{\rm{H}}}_{2}}$) and oxidation potential (${{\rm{E}}}_{{O}_{2}/{{\rm{H}}}_{2}O}$) of water are −4.44 and −5.67 eV, respectively. These values lie just within the bandgaps of the GaS, GaSe, GaTe, InS, InSe, and InTe monolayers, suggesting that these 2D materials can potentially serve as photocatalysts for water splitting, as previously reported by Zhuang *et al*.^2^.

Recent studies show that 2D-material-based Schottky contacts have great potential in nanoelectronic devices^36–38^ and sensors^39^. To explore the opportunities of forming a Schottky contact between each M_III_X monolayer and each 2D Dirac material, we calculated and obtained values of 4.23 and 4.60 eV as the the work functions of graphene and silicene, respectively. Figure 3 shows that graphene can form n-type Schottky contacts with GaS, GaSe, GaTe, and InTe, and n-type Ohmic contacts with InS and InSe. These predictions are in good agreement with results of recent studies^40^,^41^. Meanwhile, silicene can form n-type Schottky contacts with GaS, GaSe, GaTe, InS, InSe, and InTe. Overall, our findings are expected to be useful to the design of Schottky devices with dedicated Schottky barrier height.

In addition, p–n junctions are fimportant for building nanoelectronic devices. Control of the carrier type in 2D semiconducting materials is a fundamental requirement for the application of nanoelectronic devices in various fields. Peng *et al*.^42^ developed a method for direct inject the carrier into a nanochannel by using a metal electrode with proper work function. That is a p-type (or n-type) device channel can be obtained by chosing a metal electrode with higher (or lower) work function than that in the channel. Figure 3 shows the band alignment of GaS, GaSe, GaTe, InS, InSe, and InTe. The work functions of widely used metals for electrode like Y, Al, Cu, Ag, Au, and Pt are also shown for comparison^43^. Obviously, when Al and Ag are used as the electrode material, both the Al/InS and Ag/InS interfaces may form an Ohmic contact. Furthermore, when Y is used as the electrode material, all of the Y/M_III_X interfaces are highly probably form an Ohmic contacts, inducing effective carrier injection as well as enhancement of contact performance.

**Figure 4:**
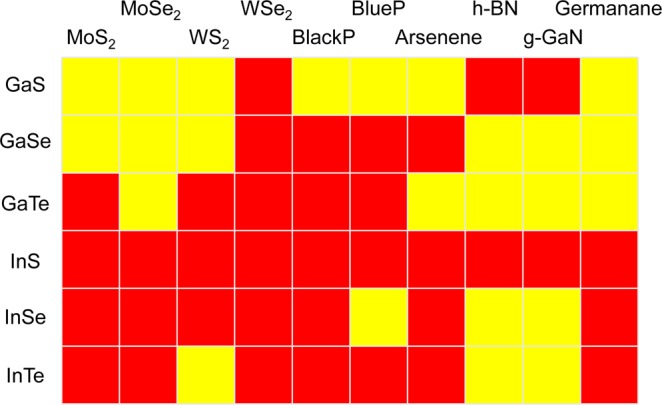
Table of heterostructures formed between M_III_Xs (GaS, GaSe, GaTe, InS, InSe, and InTe) and various 2D semiconducting materials (MoS_2_, MoSe_2_, WS_2_, WSe_2_, BlackP, BlueP, arsenene, h-BN, g-GaN, and germanane). Type-I and type-II heterostructures are shown in yellow and red, respectively.

The main obstacle to the application of a material in photocatalysis is the short life time of photo-generated carriers. Generally, forming a van der Waals (vdW) heterostructure with type-II band alignment can significantly extend the life time of photo-generated carriers^44–51^. We systematically investigated the possibility of forming type-II heterostructures by combining M_III_Xs with other popular 2D semiconducting materials, including MoS_2_, MoSe_2_, WS_2_, WSe_2_, black phosphorene (BlackP), blue phosphorene (BlueP), arsenene, h-BN, g-GaN, and germanane; the results are presented in Fig. 4. In brief, GaS can form type-II heterostructures with WSe_2_, h-BN, and g-GaN; GaSe can form type-II heterostructures with WSe_2_, BlackP, BlueP, and arsenene; GaTe can form type-II heterostructures with MoS_2_, WS_2_, WSe_2_, BlackP, and BlueP; InS can form type-II heterostructures with all of the 2D semiconducting materials examined in our study; InSe can form type-II heterostructures with MoS_2_, MoSe_2_, WS_2_, WSe_2_, BlackP, arsenene, and germanane; and InTe can form type-II heterostructures with MoS_2_, MoSe_2_, WSe_2_, BlackP, BlueP, arsenene, and germanane. These results can provide useful guidelines for designing high-efficiency M_III_X-based photocatalysts for water splitting.

Using first-principles calculations, we systematically investigated the electronic properties and band alignment of a family of 2D semiconducting materials–group III monochalcogenides (GaS, GaSe, GaTe, InS, InSe, and InTe). We found that all six M_III_X materials are indirect-bandgap semiconducting materials (the bandgaps of GaS, GaSe, GaTe, InS, InSe, and InTe are 3.29, 2.77, 2.13, 2.63, 2.30, and 2.07 eV, respectively). Interestingly, we discovered band convergence in all of the M_III_X materials, indicating their potential for thermoelectric applications. The calculated results for band alignment of the M_III_Xs indicate that all of the M_III_X monolayers are potential photocatalysts for water splitting. Moreover, the M_III_X monolayers can form type-II heterostructures with other popular 2D semiconducting materials, which is a critical requirement for photocatalyst and photovoltaic applications. We also found that most M_III_X monolayers can form n-type Schottky contacts with graphene and silicene. In addition, when elemental Y is used as the electrode material, all of the Y/M_III_X interfaces may form Ohmic contacts. We believe our findings can help to extend the application of group III monochalcogenides in thermoelectrics, photocatalysis, photovoltaics, and nanoelectronics.
